# Trichlorido(6-methyl-2,2′-bipyridine-κ^2^ *N*,*N*′)(dimethyl­sulfoxide-κ*O*)indium(III)

**DOI:** 10.1107/S1600536812041049

**Published:** 2012-10-06

**Authors:** Sadif A. Shirvan, Sara Haydari Dezfuli, Elyas Golabi, Mohammad Amin Gholamzadeh

**Affiliations:** a Department of Chemistry, Omidieh Branch, Islamic Azad University, Omidieh, Iran; b Department of Petroleum Engineering, Omidieh Branch, Islamic Azad University, Omidieh, Iran

## Abstract

In the title compound, [In(C_11_H_10_N_2_)Cl_3_(C_2_H_6_OS)], the In^III^ cation is six-coordinated in a distorted octa­hedral configuration by two N atoms from the chelating 6-methyl-2,2′-bipyridine ligand, one O atom from a dimethyl­sulfoxide group and three Cl^−^ anions. Weak inter­molecular C—H⋯O and C—H⋯Cl hydrogen bonds and intra­molecular C—H⋯Cl hydrogen bonds are present in the structure.

## Related literature

For related structures, see: Abedi *et al.* (2012*a* ▶,*b* ▶); Ahmadi *et al.* (2008*a* ▶,*b* ▶,*c* ▶, 2009 ▶); Amani *et al.* (2009 ▶); Ilyukhin *et al.* (1994 ▶); Kalateh *et al.* (2008 ▶, 2010 ▶); Malyarick *et al.* (1992 ▶); Nan *et al.* (1987 ▶); Newkome *et al.* (1982 ▶); Onggo *et al.* (1990 ▶, 2005 ▶); Shirvan & Haydari Dezfuli (2012*a* ▶,*b* ▶); Shirvan *et al.* (2012 ▶).
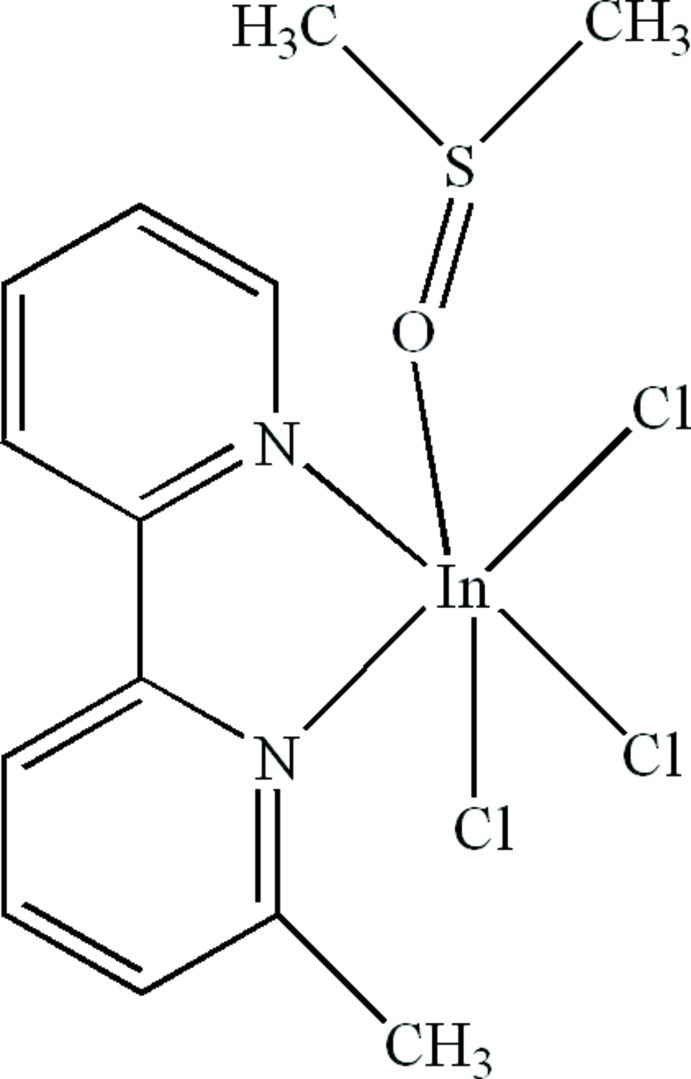


## Experimental

### 

#### Crystal data

[In(C_11_H_10_N_2_)Cl_3_(C_2_H_6_OS)]
*M* *_r_* = 469.52
Monoclinic, 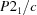

*a* = 13.0169 (6) Å
*b* = 8.5548 (3) Å
*c* = 15.9964 (8) Å
β = 93.393 (4)°
*V* = 1778.19 (14) Å^3^
*Z* = 4
Mo *K*α radiation
μ = 1.90 mm^−1^
*T* = 298 K
0.40 × 0.25 × 0.20 mm

#### Data collection

Bruker APEXII CCD area detector diffractometer
Absorption correction: multi-scan (*SADABS*; Bruker, 2001 ▶) *T*_min_ = 0.581, *T*_max_ = 0.701
14275 measured reflections
3496 independent reflections
2831 reflections with *I* > 2σ(*I*)
*R*_int_ = 0.053

#### Refinement

*R*[*F* ^2^ > 2σ(*F* ^2^)] = 0.033
*wR*(*F* ^2^) = 0.076
*S* = 1.04
3496 reflections
192 parameters
H-atom parameters constrained
Δρ_max_ = 0.65 e Å^−3^
Δρ_min_ = −0.54 e Å^−3^

### 

Data collection: *APEX2* (Bruker, 2007 ▶); cell refinement: *SAINT* (Bruker, 2007 ▶); data reduction: *SAINT*; program(s) used to solve structure: *SHELXS97* (Sheldrick, 2008 ▶); program(s) used to refine structure: *SHELXL97* (Sheldrick, 2008 ▶); molecular graphics: *SHELXTL* (Sheldrick, 2008 ▶); software used to prepare material for publication: *SHELXTL*.

## Supplementary Material

Crystal structure: contains datablock(s) I, global. DOI: 10.1107/S1600536812041049/xu5625sup1.cif

Structure factors: contains datablock(s) I. DOI: 10.1107/S1600536812041049/xu5625Isup2.hkl

Additional supplementary materials:  crystallographic information; 3D view; checkCIF report

## Figures and Tables

**Table 1:** Selected bond lengths (Å)

In1—Cl1	2.4330 (10)
In1—Cl2	2.4468 (11)
In1—Cl3	2.4309 (9)
In1—O1	2.227 (2)
In1—N1	2.270 (3)
In1—N2	2.398 (3)

**Table 2:** Hydrogen-bond geometry (Å, °)

*D*—H⋯*A*	*D*—H	H⋯*A*	*D*⋯*A*	*D*—H⋯*A*
C1—H1⋯Cl2	0.93	2.75	3.348 (5)	123
C11—H11*B*⋯Cl3	0.96	2.56	3.358 (6)	140
C13—H13*A*⋯O1^i^	0.96	2.47	3.419 (6)	169
C13—H13*C*⋯Cl2^ii^	0.96	2.82	3.612 (5)	141

Symmetry codes: (i) 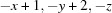; (ii) 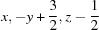.

**Fig. 1.**
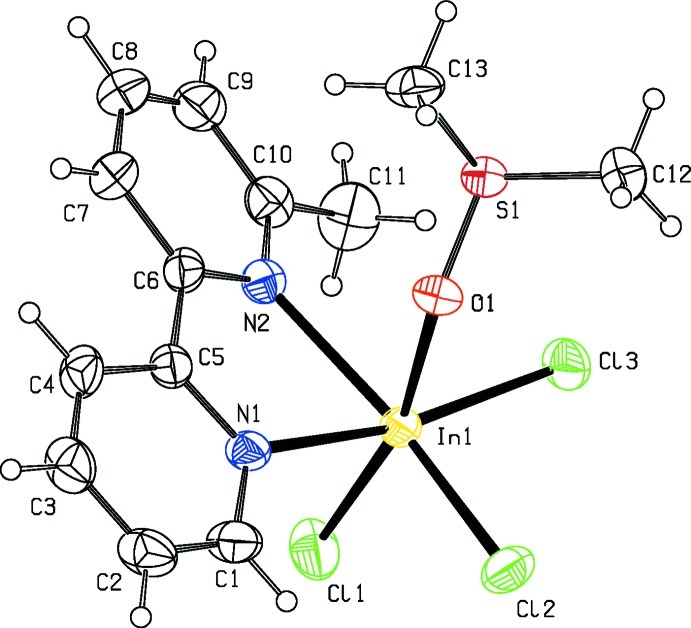
The molecular structure of the title molecule, with the atom-numbering scheme. Displacement ellipsoids are drawn at the 30% probability level.

**Fig. 2.**
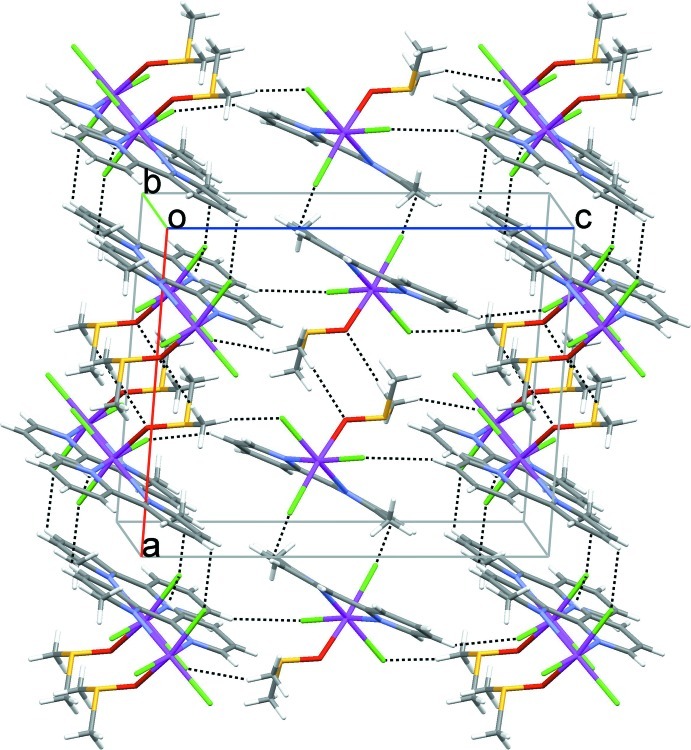
Unit-cell packing diagram for title molecule. Hydrogen bonds are shown as dashed lines.

## supplementary crystallographic information

### Comment

Recently, we reported the synthesis and crystal structure of [In(4,4'-dmbipy)Cl_3_(MeOH)].MeOH, (II), (Shirvan & Haydari Dezfuli, 2012*a*) and [In{NH(py)_2_)Cl_3_(DMSO)], (III), (Shirvan & Haydari Dezfuli, 2012*b*) [where 4,4'-dmbpy is 4,4'-dimethyl-2,2'-bipyridine and NH(py)_2_ is di-2-pyridylamine]. There are several In^III^ complexes, with formula, [In(N—N)Cl_3_(*L*)], (*L* = DMSO, H_2_O, MeOH and EtOH), such as [In(bipy)Cl_3_(H_2_O)], (IV), [In(bipy)Cl_3_(EtOH)], (V) and [In(bipy)Cl_3_(H_2_O)].H_2_O, (VI), (Malyarick *et al.*, 1992), [In(phen)Cl_3_(DMSO)], (VII), (Nan *et al.*, 1987), [In(phen)Cl_3_(H_2_O)], (VIII) and [In(phen)Cl_3_(EtOH)].EtOH, (IX), (Ilyukhin & Malyarik, 1994), [In(4,4'-dmbipy)Cl_3_(DMSO)], (*X*), (Ahmadi *et al.*, 2008*a*), [In(5,5'-dmbipy)Cl_3_(MeOH)], (XI), (Kalateh *et al.*, 2008), [In(4,4'-dtbipy)Cl_3_(MeOH)].0.5MeOH, (XII), (Abedi *et al.*, 2012*a*), [In(4 b t)Cl_3_(MeOH)], (XIII) and [In(4 b t)Cl_3_(DMSO)], (XIV), (Abedi *et al.*, 2012*b*) [where bipy is 2,2'-bipyridine, phen is 1,10-phenanthroline, DMSO is dimethyl sulfoxide, 4,4'-dmbpy is 4,4'-dimethyl-2,2'-bipyridine, 5,5'-dmbpy is 5,5'-dimethyl-2,2'-bipyridine, 4,4'-dtbipy is 4,4'-di-τert-butyl-2,2'-bipyridine and 4 b t is 4,4'-bithiazole] have been synthesized and characterized by single-crystal X-ray diffraction methods. 6-Methyl-2,2'-bipyridine (6-mbipy) is a good ligand and a few complexes with 6-mbipy have been prepared, such as that of [Hg(6-mbipy)Cl_2_], (XV), (Ahmadi *et al.*, 2008*b*), [Pt(6-mbipy)Cl_4_], (XVI), (Amani *et al.*, 2009), [Pb_4_(NO_3_)_8_(6-mbipy)_4_], (XVII), (Ahmadi *et al.*, 2009), [Zn(6-mbipy)Br_2_], (XVIII), (Kalateh *et al.*, 2010), [Zn(6-mbipy)Cl_2_], (IXX), (Ahmadi *et al.*, 2008*c*), [Pd(6-mbipy)Cl_2_], (XX), (Newkome *et al.*, 1982), [Ru(6-mbipy)_3_][BF_4_]_2_, (XXI), (Onggo *et al.*, 2005), [Fe(6-mbipy)_3_][ClO_4_]_2_.6-mbipy, (XXII), (Onggo *et al.*, 1990), and [Cd(6-mbipy)Br_2_(DMSO)], (XXIII), (Shirvan *et al.*, 2012). We report herein the synthesis and crystal structure of the title compound (I).

In the title compound, (Fig. 1), the In^III^ atom is six-coordinated in a distorted octahedral configuration by two N atoms from the chelating 6-methyl-2,2'-bipyridine ligand, one O atom from a dimethyl sulfoxide and three Cl atoms. The In—Cl, In—N and In—O bond lengths and angles are collected in Table 1.

In the crystal structure, intermolecular C—H···O and C—H···Cl hydrogen bonds and π-π contact (Table 2 & Fig. 2) between the pyridine rings, *Cg*3—*Cg*3^i^ [symmetry cods: (i) –*X*,2-Y,-*Z*, where *Cg*3 is centroid of the ring (N2/C6—C10)] may stabilize the structure, with centroid-centroid distance 3.774 (2) Å.

### Experimental

For the preparation of the title compound, (I), a solution of 6-methyl-2,2'-bipyridine (0.28 g, 0.26 ml, 1.65 mmol) in methanol (10 ml) was added to a solution of InCl_3_.4H_2_O (0.48 g, 1.65 mmol) in methanol (10 ml) at room temperature. The suitable crystals for X-ray diffraction experiment were obtained by methanol diffusion to a colorless solution in DMSO. Suitable crystals were isolated after one week (yield; 0.56 g, 72.3%).

### Refinement

All H atoms were positioned geometrically with C—H = 0.93 and 0.96 Å and constrained to ride on their parent atoms with *U*_iso_(H) = 1.5U_eq_(C) for methyl H atoms and 1.2U_eq_(C) for the others.

### Crystal data

**Table:**

[In(C_11_H_10_N_2_)Cl_3_(C_2_H_6_OS)]	*F*(000) = 928
*M**_r_* = 469.52	*D*_x_ = 1.754 Mg m^−^^3^
Monoclinic, *P*2_1_/*c*	Mo *K*α radiation, λ = 0.71073 Å
Hall symbol: -P 2ybc	Cell parameters from 14275 reflections
*a* = 13.0169 (6) Å	θ = 1.6–26.0°
*b* = 8.5548 (3) Å	µ = 1.90 mm^−^^1^
*c* = 15.9964 (8) Å	*T* = 298 K
β = 93.393 (4)°	Prism, colorless
*V* = 1778.19 (14) Å^3^	0.40 × 0.25 × 0.20 mm
*Z* = 4	

### Data collection

**Table:**

Bruker APEXII CCD area detector diffractometer	3496 independent reflections
Radiation source: fine-focus sealed tube	2831 reflections with *I* > 2σ(*I*)
Graphite monochromator	*R*_int_ = 0.053
ω scans	θ_max_ = 26.0°, θ_min_ = 1.6°
Absorption correction: multi-scan (*SADABS*; Bruker, 2001)	*h* = −16→15
*T*_min_ = 0.581, *T*_max_ = 0.701	*k* = −10→10
14275 measured reflections	*l* = −17→19

### Refinement

**Table:**

Refinement on *F*^2^	Primary atom site location: structure-invariant direct methods
Least-squares matrix: full	Secondary atom site location: difference Fourier map
*R*[*F*^2^ > 2σ(*F*^2^)] = 0.033	Hydrogen site location: inferred from neighbouring sites
*wR*(*F*^2^) = 0.076	H-atom parameters constrained
*S* = 1.04	*w* = 1/[σ^2^(*F*_o_^2^) + (0.042*P*)^2^] where *P* = (*F*_o_^2^ + 2*F*_c_^2^)/3
3496 reflections	(Δ/σ)_max_ = 0.013
192 parameters	Δρ_max_ = 0.65 e Å^−^^3^
0 restraints	Δρ_min_ = −0.54 e Å^−^^3^

### Special details

**Table:**

Geometry. All e.s.d.'s (except the e.s.d. in the dihedral angle between two l.s. planes) are estimated using the full covariance matrix. The cell e.s.d.'s are taken into account individually in the estimation of e.s.d.'s in distances, angles and torsion angles; correlations between e.s.d.'s in cell parameters are only used when they are defined by crystal symmetry. An approximate (isotropic) treatment of cell e.s.d.'s is used for estimating e.s.d.'s involving l.s. planes.
Refinement. Refinement of *F*^2^ against ALL reflections. The weighted *R*-factor *wR* and goodness of fit *S* are based on *F*^2^, conventional *R*-factors *R* are based on *F*, with *F* set to zero for negative *F*^2^. The threshold expression of *F*^2^ > σ(*F*^2^) is used only for calculating *R*-factors(gt) *etc*. and is not relevant to the choice of reflections for refinement. *R*-factors based on *F*^2^ are statistically about twice as large as those based on *F*, and *R*- factors based on ALL data will be even larger.

### Fractional atomic coordinates and isotropic or equivalent isotropic displacement parameters (Å^2^)

**Table:**

	*x*	*y*	*z*	*U*_iso_*/*U*_eq_	
N1	0.2514 (2)	0.9449 (3)	0.13539 (18)	0.0467 (7)	
Cl1	0.12219 (10)	0.60548 (12)	0.13692 (9)	0.0801 (4)	
C11	0.0925 (4)	0.6719 (6)	−0.1127 (4)	0.0878 (16)	
H11A	0.0557	0.6185	−0.0709	0.105*	
H11B	0.1589	0.6247	−0.1165	0.105*	
H11C	0.0545	0.6644	−0.1658	0.105*	
Cl2	0.40731 (9)	0.63682 (14)	0.17190 (7)	0.0715 (3)	
N2	0.1563 (2)	0.8750 (3)	−0.01655 (19)	0.0455 (7)	
C5	0.2065 (3)	1.0631 (4)	0.0914 (2)	0.0453 (8)	
C2	0.2834 (4)	1.1141 (5)	0.2520 (3)	0.0704 (12)	
H2	0.3095	1.1284	0.3069	0.084*	
C3	0.2403 (4)	1.2359 (5)	0.2066 (3)	0.0676 (12)	
H3	0.2373	1.3349	0.2304	0.081*	
In1	0.269337 (19)	0.71247 (3)	0.069351 (15)	0.04247 (9)	
C6	0.1608 (3)	1.0271 (4)	0.0064 (2)	0.0458 (8)	
Cl3	0.30194 (9)	0.49559 (11)	−0.02363 (7)	0.0630 (3)	
S1	0.38648 (7)	0.84709 (11)	−0.09440 (5)	0.0444 (2)	
C9	0.0639 (3)	0.9526 (7)	−0.1425 (3)	0.0697 (12)	
H9	0.0300	0.9247	−0.1932	0.084*	
C12	0.5099 (4)	0.7693 (6)	−0.1071 (3)	0.0766 (13)	
H12C	0.5248	0.7738	−0.1651	0.092*	
H12B	0.5121	0.6626	−0.0885	0.092*	
H12A	0.5602	0.8292	−0.0745	0.092*	
C10	0.1050 (3)	0.8364 (5)	−0.0894 (3)	0.0572 (10)	
C13	0.4079 (4)	1.0435 (5)	−0.1239 (3)	0.0660 (11)	
H13A	0.4648	1.0855	−0.0902	0.079*	
H13B	0.3473	1.1042	−0.1157	0.079*	
H13C	0.4232	1.0472	−0.1819	0.079*	
C8	0.0727 (4)	1.1049 (7)	−0.1215 (3)	0.0752 (13)	
H8	0.0467	1.1820	−0.1579	0.090*	
C1	0.2870 (4)	0.9711 (5)	0.2141 (2)	0.0635 (11)	
H1	0.3156	0.8878	0.2448	0.076*	
C7	0.1207 (3)	1.1444 (5)	−0.0456 (3)	0.0668 (11)	
H7	0.1262	1.2486	−0.0293	0.080*	
O1	0.37807 (18)	0.8589 (3)	0.00075 (14)	0.0470 (6)	
C4	0.2017 (3)	1.2113 (4)	0.1264 (3)	0.0581 (10)	
H4	0.1722	1.2935	0.0954	0.070*	

### Atomic displacement parameters (Å^2^)

**Table:**

	*U*^11^	*U*^22^	*U*^33^	*U*^12^	*U*^13^	*U*^23^
N1	0.0648 (19)	0.0362 (15)	0.0397 (16)	−0.0001 (13)	0.0090 (13)	0.0021 (13)
Cl1	0.0865 (8)	0.0494 (6)	0.1099 (9)	−0.0006 (5)	0.0514 (7)	0.0107 (6)
C11	0.080 (3)	0.086 (4)	0.093 (4)	−0.007 (3)	−0.024 (3)	−0.025 (3)
Cl2	0.0945 (8)	0.0633 (6)	0.0549 (6)	0.0203 (6)	−0.0109 (5)	0.0082 (5)
N2	0.0420 (15)	0.0475 (17)	0.0473 (17)	−0.0019 (13)	0.0036 (13)	0.0010 (13)
C5	0.0477 (19)	0.0385 (18)	0.051 (2)	−0.0012 (15)	0.0133 (16)	0.0036 (15)
C2	0.105 (3)	0.059 (3)	0.048 (2)	−0.010 (2)	0.010 (2)	−0.010 (2)
C3	0.088 (3)	0.044 (2)	0.072 (3)	−0.004 (2)	0.018 (2)	−0.013 (2)
In1	0.05532 (16)	0.03124 (13)	0.04150 (14)	0.00100 (11)	0.00834 (10)	0.00223 (10)
C6	0.0443 (18)	0.0391 (18)	0.055 (2)	0.0020 (14)	0.0118 (16)	0.0051 (16)
Cl3	0.0866 (7)	0.0446 (5)	0.0588 (6)	0.0033 (5)	0.0124 (5)	−0.0110 (4)
S1	0.0469 (5)	0.0475 (5)	0.0386 (4)	−0.0044 (4)	0.0013 (4)	0.0013 (4)
C9	0.053 (2)	0.102 (4)	0.053 (2)	0.010 (2)	−0.0027 (19)	0.002 (3)
C12	0.072 (3)	0.092 (3)	0.067 (3)	0.027 (3)	0.017 (2)	0.000 (3)
C10	0.046 (2)	0.069 (3)	0.056 (2)	−0.0017 (18)	0.0019 (18)	−0.007 (2)
C13	0.094 (3)	0.056 (2)	0.048 (2)	−0.006 (2)	0.004 (2)	0.0128 (19)
C8	0.079 (3)	0.088 (4)	0.058 (3)	0.024 (3)	0.000 (2)	0.017 (2)
C1	0.099 (3)	0.050 (2)	0.042 (2)	−0.002 (2)	0.006 (2)	0.0023 (17)
C7	0.079 (3)	0.057 (2)	0.065 (3)	0.016 (2)	0.009 (2)	0.014 (2)
O1	0.0514 (14)	0.0532 (14)	0.0367 (13)	−0.0085 (11)	0.0046 (10)	0.0020 (11)
C4	0.067 (2)	0.0359 (18)	0.073 (3)	0.0062 (18)	0.010 (2)	−0.0004 (19)

### Geometric parameters (Å, º)

**Table:**

In1—Cl1	2.4330 (10)	C3—H3	0.9300
In1—Cl2	2.4468 (11)	C6—C7	1.385 (5)
In1—Cl3	2.4309 (9)	S1—O1	1.536 (2)
In1—O1	2.227 (2)	S1—C12	1.762 (4)
In1—N1	2.270 (3)	S1—C13	1.772 (4)
In1—N2	2.398 (3)	C9—C8	1.348 (7)
N1—C1	1.335 (5)	C9—C10	1.394 (6)
N1—C5	1.346 (4)	C9—H9	0.9300
C11—C10	1.462 (6)	C12—H12C	0.9600
C11—H11A	0.9600	C12—H12B	0.9600
C11—H11B	0.9600	C12—H12A	0.9600
C11—H11C	0.9600	C13—H13A	0.9600
N2—C10	1.349 (5)	C13—H13B	0.9600
N2—C6	1.353 (4)	C13—H13C	0.9600
C5—C4	1.390 (5)	C8—C7	1.376 (7)
C5—C6	1.483 (5)	C8—H8	0.9300
C2—C1	1.368 (6)	C1—H1	0.9300
C2—C3	1.371 (7)	C7—H7	0.9300
C2—H2	0.9300	C4—H4	0.9300
C3—C4	1.367 (7)		
			
C1—N1—C5	118.9 (3)	N2—C6—C5	117.2 (3)
C1—N1—In1	123.2 (3)	C7—C6—C5	121.1 (3)
C5—N1—In1	117.9 (2)	O1—S1—C12	104.9 (2)
C10—C11—H11A	109.5	O1—S1—C13	102.90 (18)
C10—C11—H11B	109.5	C12—S1—C13	99.7 (2)
H11A—C11—H11B	109.5	C8—C9—C10	120.9 (4)
C10—C11—H11C	109.5	C8—C9—H9	119.6
H11A—C11—H11C	109.5	C10—C9—H9	119.6
H11B—C11—H11C	109.5	S1—C12—H12C	109.5
C10—N2—C6	118.8 (3)	S1—C12—H12B	109.5
C10—N2—In1	127.5 (3)	H12C—C12—H12B	109.5
C6—N2—In1	112.8 (2)	S1—C12—H12A	109.5
N1—C5—C4	120.2 (4)	H12C—C12—H12A	109.5
N1—C5—C6	117.5 (3)	H12B—C12—H12A	109.5
C4—C5—C6	122.2 (3)	N2—C10—C9	120.3 (4)
C1—C2—C3	117.9 (4)	N2—C10—C11	119.8 (4)
C1—C2—H2	121.0	C9—C10—C11	119.9 (4)
C3—C2—H2	121.0	S1—C13—H13A	109.5
C4—C3—C2	119.8 (4)	S1—C13—H13B	109.5
C4—C3—H3	120.1	H13A—C13—H13B	109.5
C2—C3—H3	120.1	S1—C13—H13C	109.5
O1—In1—N1	79.77 (10)	H13A—C13—H13C	109.5
O1—In1—N2	77.17 (9)	H13B—C13—H13C	109.5
N1—In1—N2	71.48 (10)	C9—C8—C7	119.0 (4)
O1—In1—Cl3	89.36 (7)	C9—C8—H8	120.5
N1—In1—Cl3	168.44 (8)	C7—C8—H8	120.5
N2—In1—Cl3	102.44 (8)	N1—C1—C2	123.3 (4)
O1—In1—Cl1	166.02 (7)	N1—C1—H1	118.3
N1—In1—Cl1	91.00 (8)	C2—C1—H1	118.3
N2—In1—Cl1	90.00 (7)	C8—C7—C6	119.2 (4)
Cl3—In1—Cl1	98.93 (4)	C8—C7—H7	120.4
O1—In1—Cl2	90.94 (7)	C6—C7—H7	120.4
N1—In1—Cl2	90.64 (8)	S1—O1—In1	122.40 (14)
N2—In1—Cl2	159.89 (8)	C3—C4—C5	119.7 (4)
Cl3—In1—Cl2	93.47 (4)	C3—C4—H4	120.1
Cl1—In1—Cl2	99.71 (5)	C5—C4—H4	120.1
N2—C6—C7	121.7 (4)		
			
C1—N1—C5—C4	2.8 (5)	In1—N2—C6—C5	−17.8 (4)
In1—N1—C5—C4	−174.4 (3)	N1—C5—C6—N2	7.9 (5)
C1—N1—C5—C6	−175.5 (3)	C4—C5—C6—N2	−170.3 (3)
In1—N1—C5—C6	7.3 (4)	N1—C5—C6—C7	−174.6 (3)
C1—C2—C3—C4	0.6 (7)	C4—C5—C6—C7	7.1 (5)
C1—N1—In1—O1	−109.3 (3)	C6—N2—C10—C9	4.4 (5)
C5—N1—In1—O1	67.8 (2)	In1—N2—C10—C9	−163.4 (3)
C1—N1—In1—N2	170.9 (3)	C6—N2—C10—C11	−175.5 (4)
C5—N1—In1—N2	−12.0 (2)	In1—N2—C10—C11	16.7 (5)
C1—N1—In1—Cl3	−129.4 (4)	C8—C9—C10—N2	−1.3 (6)
C5—N1—In1—Cl3	47.8 (6)	C8—C9—C10—C11	178.6 (5)
C1—N1—In1—Cl1	81.2 (3)	C10—C9—C8—C7	−1.7 (7)
C5—N1—In1—Cl1	−101.6 (2)	C5—N1—C1—C2	−2.3 (6)
C1—N1—In1—Cl2	−18.5 (3)	In1—N1—C1—C2	174.8 (3)
C5—N1—In1—Cl2	158.7 (2)	C3—C2—C1—N1	0.6 (7)
C10—N2—In1—O1	100.7 (3)	C9—C8—C7—C6	1.5 (7)
C6—N2—In1—O1	−67.7 (2)	N2—C6—C7—C8	1.7 (6)
C10—N2—In1—N1	−176.0 (3)	C5—C6—C7—C8	−175.6 (4)
C6—N2—In1—N1	15.6 (2)	C12—S1—O1—In1	−112.8 (2)
C10—N2—In1—Cl3	14.2 (3)	C13—S1—O1—In1	143.3 (2)
C6—N2—In1—Cl3	−154.2 (2)	N1—In1—O1—S1	−139.25 (18)
C10—N2—In1—Cl1	−84.9 (3)	N2—In1—O1—S1	−66.10 (17)
C6—N2—In1—Cl1	106.7 (2)	Cl3—In1—O1—S1	36.81 (16)
C10—N2—In1—Cl2	155.7 (2)	Cl1—In1—O1—S1	−89.9 (3)
C6—N2—In1—Cl2	−12.7 (4)	Cl2—In1—O1—S1	130.27 (16)
C10—N2—C6—C7	−4.7 (5)	C2—C3—C4—C5	−0.1 (7)
In1—N2—C6—C7	164.9 (3)	N1—C5—C4—C3	−1.7 (6)
C10—N2—C6—C5	172.7 (3)	C6—C5—C4—C3	176.5 (4)

### Hydrogen-bond geometry (Å, º)

**Table:**

*D*—H···*A*	*D*—H	H···*A*	*D*···*A*	*D*—H···*A*
C1—H1···Cl2	0.93	2.75	3.348 (5)	123
C11—H11*B*···Cl3	0.96	2.56	3.358 (6)	140
C13—H13*A*···O1^i^	0.96	2.47	3.419 (6)	169
C13—H13*C*···Cl2^ii^	0.96	2.82	3.612 (5)	141

Symmetry codes: (i) −*x*+1, −*y*+2, −*z*; (ii) *x*, −*y*+3/2, *z*−1/2.

## References

[bb1] Abedi, A., Safari, A. R. & Amani, V. (2012*a*). *Z. Kristallogr. New Cryst. Struct.* **227**, 169–198.

[bb2] Abedi, A., Safari, N., Amani, V. & Khavasi, H. R. (2012*b*). *J. Coord. Chem.* **65**, 325–338.

[bb3] Ahmadi, R., Ebadi, A., Kalateh, K., Norouzi, A. & Amani, V. (2008*b*). *Acta Cryst.* E**64**, m1407. 10.1107/S1600536808032777 PMC2959542 21580857

[bb4] Ahmadi, R., Kalateh, K., Abedi, A., Amani, V. & Khavasi, H. R. (2008*c*). *Acta Cryst.* E**64**, m1306–m1307. 10.1107/S1600536808029553 PMC2959453 21201045

[bb5] Ahmadi, R., Kalateh, K., Alizadeh, R., Khoshtarkib, Z. & Amani, V. (2009). *Acta Cryst.* E**65**, m1169–m1170. 10.1107/S160053680903459X PMC2970246 21577706

[bb6] Ahmadi, R., Kalateh, K., Ebadi, A., Amani, V. & Khavasi, H. R. (2008*a*). *Acta Cryst.* E**64**, m1266. 10.1107/S1600536808028894 PMC2959229 21201019

[bb7] Amani, V., Safari, N., Khavasi, H. R. & Akkurt, M. (2009). *Polyhedron*, **28**, 3026–3030.

[bb8] Bruker (2001). *SADABS* Bruker AXS Inc., Madison, Wisconsin, USA.

[bb9] Bruker (2007). *APEX2* and *SAINT* Bruker AXS Inc., Madison, Wisconsin, USA.

[bb10] Ilyukhin, A. B. & Malyarik, M. A. (1994). *Kristallografiya*, **39**, 439–443.

[bb11] Kalateh, K., Ahmadi, R. & Amani, V. (2010). *Acta Cryst.* E**66**, m1241. 10.1107/S1600536810035658 PMC2983320 21587391

[bb12] Kalateh, K., Ahmadi, R., Ebadi, A., Amani, V. & Khavasi, H. R. (2008). *Acta Cryst.* E**64**, m1353–m1354. 10.1107/S160053680803119X PMC2959790 21580816

[bb13] Malyarick, M. A., Petrosyants, S. P. & Ilyuhin, A. B. (1992). *Polyhedron*, **11**, 1067–1073.

[bb14] Nan, D., Naidong, W., Zhenchao, D. & Shengzhi, H. (1987). *Jiegou Huaxue*, **6**, 145–149.

[bb15] Newkome, G. R., Fronczek, F. R., Gupta, V. K., Puckett, W. E., Pantaleo, D. C. & Kiefer, G. E. (1982). *J. Am. Chem. Soc.* **104**, 1782–1783.

[bb16] Onggo, D., Hook, J. M., Rae, A. D. & Goodwin, H. A. (1990). *Inorg. Chim. Acta*, **173**, 19–30.

[bb17] Onggo, D., Scudder, M. L., Craig, D. C. & Goodwin, H. A. (2005). *J. Mol. Struct.* **738**, 129–136.

[bb18] Sheldrick, G. M. (2008). *Acta Cryst.* A**64**, 112–122. 10.1107/S0108767307043930 18156677

[bb19] Shirvan, S. A. & Haydari Dezfuli, S. (2012*a*). *Acta Cryst.* E**68**, m1189–m1190. 10.1107/S1600536812035490 PMC3435608 22969481

[bb20] Shirvan, S. A. & Haydari Dezfuli, S. (2012*b*). *Acta Cryst.* E**68**, m1124. 10.1107/S1600536812033168 PMC3414166 22904773

[bb21] Shirvan, S. A., Haydari Dezfuli, S. & Golabi, E. (2012). *Acta Cryst.* E**68**, m1256. 10.1107/S1600536812038147 PMC3470143 23125587

